# Health system preparedness in infectious diseases: perspective of Malaysia, a middle-income country, in the face of monkeypox outbreaks

**DOI:** 10.1186/s41182-022-00479-4

**Published:** 2022-11-21

**Authors:** Chang Chee Tao, Xin-Jie Lim, Awatef Amer Nordin, Chern Choong Thum, Sondi Sararaks, Kalaiarasu Periasamy, Philip Rajan

**Affiliations:** 1 Clinical Research Centre (CRC), Hospital Raja Permaisuri Bainun, Ministry of Health Malaysia, 30450 Ipoh, Perak Malaysia; 2 School of Pharmacy, Monash University Malaysia, Bandar Sunway, Malaysia; 3 Institute for Health Systems Research, National Institutes of Health, Ministry of Health Malaysia, Setia Alam, Malaysia; 4 Department of Psychiatry, Universiti Putra Malaysia Teaching Hospital, Serdang, Malaysia; 5 Institute for Clinical Research, National Institutes of Health, Ministry of Health Malaysia, Setia Alam, Malaysia

**Keywords:** Health system preparedness, Monkeypox, Infectious diseases, Malaysia

## Abstract

The World Health Organization declared monkeypox as a Public Health Emergency of International Concern on July 23, 2022. As of July 25th, 2022, there were 16,016 laboratory-confirmed cases reported worldwide with 5 deaths. Malaysia's Health Ministry has developed a five-point strategy to prepare for the impending threat of the infectious disease, encompassing early detection of monkeypox, consolidation of laboratory diagnostic facilities, case management and treatment, cluster management, and strengthening public awareness. Crisis and disaster preparedness within a nation's health system is paramount to preventing disease spread. Various strategies for developing resilience in the face of global infectious disease spread were discussed. The current disease preparedness and response framework and guidelines in Malaysia have established a health system that is proactive and responsive to any potential infectious disease outbreaks. Despite this, the future remains unpredictable, and ongoing fortification is required as events unfold.

## Introduction

On July 23, 2022, the World Health Organization (WHO) declared monkeypox a Public Health Emergency of International Concern (PHEIC)[1]. There were 76,806 laboratory-confirmed cases worldwide as of October 28th, 2022, with 36 deaths [2]. Monkeypox is an infectious disease which spreads to humans through close contact with infected animals and can also spread between humans via bodily fluids, respiratory droplets, and contaminated materials. The lack of travel history to endemic regions suggests asymptomatic community-to-community transmission and increased virus virulence [3]. In non-endemic countries, one case of monkeypox is considered an outbreak [4]. This is worrying as an unexposed population may have non-existent immunity to the virus [5]. In addition to that, these monkeypox cases were discovered to have atypical clinical presentations, implying that there may be new characteristics of the disease that are yet unknown [6]. At present, monkeypox has been declared to be a PHEIC, afflicting 102 territories worldwide, with the Western Pacific region at low–moderate risk [7].

## Lessons from COVID-19 on public health measures

Many countries, including Malaysia, have gained valuable experience from the Coronavirus Disease (COVID-19) pandemic in developing their health system preparedness and response mechanisms. Some of the most important lessons from the COVID-19 pandemic are that early warning is insufficient. In fact, it is political will and leadership, clear and consistent communication based on the evolving scientific evidence, and rapid implementation of public health measures that may make a huge difference between a runaway pandemic and a manageable outbreak [8]. Underinvestment in public health interventions and a failure to deploy public health as the first line of defence has resulted in preventable deaths [8]. As the current monkeypox outbreak has featured atypical clinical manifestations as well as rapid human-to-human transmissions, public health measures to control infection sources, block transmission routes, and protect vulnerable populations are critical [9]. The COVID-19 pandemic has undeniably proved the importance of leveraging technology in not just boosting the speed and reach of risk communication but also in providing care [10].

## Malaysia’s capacity in monkeypox outbreak preparedness and response

**Fig. 1:**
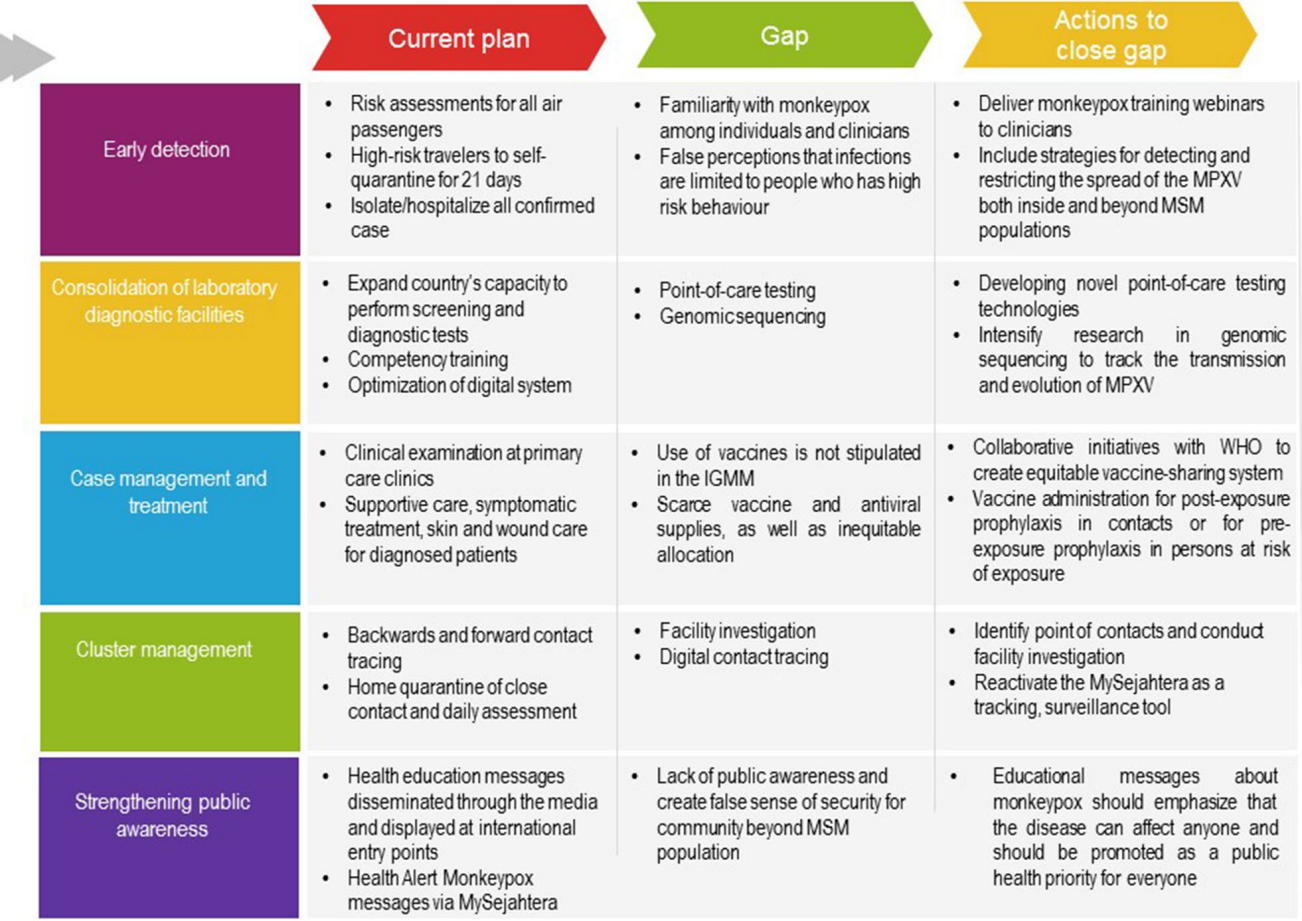
Five pillars in control and containment monkeypox outbreak: current plan, gap and action to close gap

Malaysia reported its monkeypox risk assessment findings on May 21, 2022, concluding that human-to-human transmission is limited and the risk of transmission into Malaysia is low [4]. Malaysia's Ministry of Health (MOH) has issued Interim Guidelines on Monkeypox Management (IGMM), which include a five-point strategy to combat the risk of monkeypox spreading to Malaysia (Fig. 1) [11]. These guidelines were issued prior to the release of the WHO's Technical Brief (interim) and Priority Actions document on improving monkeypox readiness in the WHO South-East Asia Region [12]. The WHO's Technical Brief (interim) and Priority Actions document identified surveillance, laboratory testing, clinical management and infection prevention and control, vaccination, and risk communication and community engagement as five management priorities for monkeypox. Similar considerations are reflected in the IGMM strategies [13].

### Five pillars in the control and containment of the monkeypox outbreak: current state, gap and action to close gap

#### Early detection of monkeypox

First, the WHO surveillance case definition of monkeypox outbreak non-endemic countries is adopted. This requirement is critical for early detection, especially for diseases unknown in the local setting, because a standardised set of criteria would allow all health providers to identify potential cases that require further investigation. Cases that have been "discarded" are included in the definition because they are no longer a potential source of disease transmission.

To reduce imported cases, risk assessment and screening at international entry points are necessary [14]. The IGMM mandates risk assessments for all air passengers and cards instructing high-risk travellers to self-quarantine for 21 days [11]. This includes cruise passengers too. Meanwhile, fever screening counters at clinics are instructed to be alert to monkeypox-like symptom presentations. All confirmed cases must be isolated, and children, pregnant women, older adults, and the immunocompromised who are at risk of severe illness are to be admitted to the hospital for observation [15].

Nonetheless, there are several factors contributing to case detection. This includes poor familiarity with monkeypox among individuals and clinicians, false perceptions that infections are limited to men who have sex with men (MSM) populations, and stigma [16]. It is crucial for health authorities to act swiftly with partners across the sector to deliver monkeypox training webinars to clinicians to raise knowledge and awareness of this virus, especially as monkeypox is uncommon in the Malaysian clinical setting. This may assist case identification at outpatient clinics, prevent undetected transmission among patients, and may be essential in public advice dissemination. Furthermore, public health interventions must include strategies for detecting and restricting the spread of the monkeypox virus (MPXV) both within and beyond MSM populations. Public health communication must include comprehensive, non-stigmatising information for the MSM population to recognise and seek treatment for MPXV symptoms and reduce transmission risks during sexual interactions [16].

#### Consolidation of laboratory diagnostic facilities

Limited MPXV testing impedes case detection and control efforts [16]. Effective clinical testing is required for accurate case identification. An assessment of the Malaysian laboratory preparedness was performed, and it was discovered the country had adequate capacity to perform screening and diagnostic tests, specifically at the Institute for Medical Research (IMR) and the National Public Health Laboratory (MKAK). Furthermore, a multi-sectoral approach was taken, confirming that laboratories of the Department of Wildlife Protection and National Parks of Peninsular Malaysia (PERHILITAN) have the capacity to conduct similar tests on animals. This allows a more robust response in the event of human-to-human transmission. This represents an important development, but it is not sufficient.

COVID-19 highlighted the value of point-of-need testing in ensuring quick test turnaround, prompt isolation, and contact tracing, therefore allowing patients to estimate their own risks. If the capacity-demand mismatch persists, monkeypox, despite being a much less transmissible and thus more readily containable virus, may become a public health threat. Rapid expansion of nationwide testing capacity is indicated for the monkeypox outbreak. A framework for rapidly distributing laboratory diagnostic tests, standardising reagents used, setting up standard operating procedures, as well as training laboratory employees, will help to facilitate seamless communication throughout routine and emergency responses. Commercial and academic diagnostic test developers may be able to expand testing capacity in public health and commercial laboratories by inventing novel testing technologies, such as point-of-need tests and tests that utilise diverse specimen types, such as saliva and urine.

#### Case management and treatment

In severe cases, monkeypox causes fever, rash, skin lesions, lymphadenopathy, and secondary bacterial infections [17]. First-line management includes supportive care and symptomatic treatment, optimal nutritional support, and fluid–electrolyte balance monitoring [15]. Skin and wound care were prioritised because skin lesions are common [12, 17]. Meanwhile, the safety and efficacy of smallpox vaccine and monkeypox treatment options such as cidofovir, tecovirimat, and vaccinia immune globulin for clinical use are being evaluated on a regular basis [14]. These include tecovirimat, which may reduce hospitalisation length [17], and vaccines that may reduce disease severity [18]. The management guidelines are frequently updated to reflect emerging evidence.

During the coronavirus pandemic, scarce vaccine and antiviral supplies, as well as inequitable allocation, hampered national and global efforts [19]. Millions of vaccines have been ordered by wealthy countries to combat monkeypox within their borders, but none have publicly committed to sharing these supplies with Africa [20, 21]. Therefore, the World Health Organization announced the creation of a new vaccine-sharing system to halt the spread of monkeypox in more than 30 countries outside of Africa [22]. The monkeypox vaccine, according to WHO recommendations [23], should only be considered for post-exposure prophylaxis in contacts such as domestic, sexual, and other contacts of community cases, as well as health workers, where there may have been a breach of individual safety, or for pre-exposure prophylaxis in persons at risk of exposure, which includes high-risk health workers, clinical laboratory personnel performing monkeypox diagnostic testing, and communities with high-risk behaviour (Table 1). Failure to provide equitable access would further erode confidence in the international system. Countries where MPXV has been common for decades are underscoring the failure to mobilise crucial medical resources in response to outbreaks in Africa. Consequently, ensuring equitable access to immunisations to prevent the most susceptible individuals from serious illness is crucial. While vaccines are not currently recommended in the Malaysian IGMM, the Ministry of Health evaluates it on a regular basis based on the most recent evidence and disease outbreak phases in the country.

**Table 1:** Vaccination policies recommendations in United Kingdom, United States and World Health Organization

Characteristic/country	UK [38]	US [39]	WHO [23]
Brand name	JYNNEOS	JYNNEOS and ACAM2000	JYNNEOS and ACAM2000
Pre-exposure vaccination	Prioritised for GBMSM at highest risk	People with certain risk factors but without confirmed exposure	Prioritised for community at higher risk such as MSM
Occupational vaccination	Prioritised for workers at high risk of exposure	Prioritised for workers at high risk of exposure	Prioritised for workers at high risk of exposure
Post exposure vaccination	Within 4 days of exposure, or up to 14 days in those who are at higher risk of complications	Within 4 days of exposure, or up to 14 days	Within four days of first exposure, and up to 14 days in the absence of symptoms

#### Cluster management

Despite the fact that there were no monkeypox cases in Malaysia at the time of writing, the IGMM [15] outlines source investigation measures such as backward and forward contact tracing and active and passive monitoring of close contacts. Home quarantine for 21 days from the last day of exposure, supplemented by daily assessments using Malaysia's contact tracing application (MySejahtera), previously developed for COVID-19 surveillance, as well as referrals of symptomatic close contacts for isolation and examination, is critical. The hotspot tracking feature on MySejahtera detects active infectious cases in the community. All visitors planning to enter Malaysia must download the application, which allows for active and passive surveillance. Notably, the Malaysian population's usage of MySejahtera decreased significantly as the perception that the coronavirus no longer poses a threat to public health spread [24].

#### Strengthening public awareness

At this stage, the Malaysian National Crisis Preparedness and Response Center (CPRC) had prepared and disseminated risk communication to the public in the form of a health advisory for travelers. Potential subjects were advised to seek treatment at the nearest healthcare facility if they became ill. The importance of self-isolation and contact tracing was provided too. Since household contact and nosocomial infection are the most common modes of transmission, they must be prioritised in public awareness programmes [17].

Public education is a critical prevention strategy for monkeypox [25]. This includes providing the public information on early treatment seeking, proper PPE usage, good hygiene practices, safe animal handling, and self-isolation [15]. This information has been disseminated through the media and is displayed at international entry points [15]. All inbound travellers from countries where monkeypox cases have been reported will receive Health Alert Monkeypox messages via MySejahtera [26]. However, educational messages about monkeypox should emphasise that the disease can affect anyone and should be promoted as a public health priority for everyone [27]. Focusing on cases of high-risk behaviour in a certain community may unfairly stigmatise this group and give people outside of the group a false sense of security.

Beyond mere awareness is the essential component of community empowerment; this eludes the role of the individual and the responsibility of controlling a possible outbreak. In moving towards endemicity of COVID-19, the success of planned strategies relies upon the concept of community solidarity and responsibility, from acquiring understanding of the disease, self-testing, and self-quarantine where necessary, and maintaining measures that prevent the spread of the disease [28]. In an infectious disease like monkeypox, the role of the empowered individual and community solidarity is just as critical.

## Moving forward

### Developing global resilience

The monkeypox outbreak has focused attention on the interface of human–animal disease transmission as well as the context in which the emergence and re-emergence of new and old infectious diseases has been widely linked to poverty and vulnerability, malnutrition, and poor healthcare behaviour [29]. Prior to its 2003 outbreak in the United States, the disease was thought to be geographically limited [30]. Developments in time and technology have now created a nearly borderless world, with disease importation via travel a significant route of disease transmission. This borderless world also calls for careful planning of strategies and health systems that are ever-evolving. Governance and strategic frameworks for response are essential components which are time-tested; incorporation of approaches leveraging on the various technologies available today can facilitate swift implementation of actions. However, this is dependent on digital literacy, with vulnerable groups at risk of being left behind.

Tambo and Al-Nazawi argued for preparedness and response resilience through methods such as effective mitigating and information communication against social media rumours and misinformation, as well as identifying research priorities in monkeypox to address existing knowledge gaps [29]. In addition, it is crucial to reexamine socioeconomic determinants of health, such as poverty and marginalisation, which are associated with an increased risk of disease, limited access to health care, and poorer health outcomes.

### Psychological needs and research

It is possible that the response framework could be further strengthened by incorporating a psychological component and conducting research on innovative treatment options. It is important to take preventative measures in order to reduce the negative social connotations associated with the disease, particularly among sexual minorities [31]. As a result of patients with mood disorders being hospitalised for longer periods of time, clinical psychology interventions may be necessary [17]. In addition, it is immediately necessary to conduct large-scale clinical tests and research on novel drugs and vaccines in both adults and children in order to demonstrate that they are safe and effective. Patients who are prescribed novel therapeutic agents require intensive monitoring and counselling regarding the likelihood of experiencing adverse effects [17]. The introduction of point-of-care testing could be the key to making rapid diagnosis of the monkeypox virus possible in rural areas [32].

### Monkeypox as a PHEIC and brief situation in Malaysia

Following the second meeting of the International Health Regulations (2005) Emergency Committee on July 21^st^, 2022, WHO declared monkeypox a PHEIC [23]. In four days, laboratory capacities in Malaysia were increased from 2 to 12 (8 public and 4 public laboratories). Information was disseminated to the public on increasing surveillance and the requirement of urgent notification by health providers if monkeypox cases were suspected or diagnosed [33]. As Monkeypox health alerts were activated earlier in June 2020 via the MySejahtera application, risk communication for travellers had already begun, and travellers to Malaysia could be monitored for the development of Monkeypox symptoms simultaneously with ongoing COVID-19 surveillance. In the first 3 weeks of July, approximately 500,000 travellers arrived in Malaysia from countries reporting monkeypox cases and were sent health alerts via MySejahtera. A case in Singapore involving a Malaysian man who visited Johor Bahru was followed through with a contact tracing of all contacts, all of whom were confirmed negative [34]. As of July 23, 2022, 9 cases have been notified to the MOH, and all were confirmed to be negative [33]. As the country borders several other countries, with cases reported in Thailand, Indonesia, and Singapore [35], surveillance at country entry points, including land borders, is being increased through collaborative efforts between Malaysia and Thailand in monitoring common borders in the north of Malaysia [36].

## Conclusion

The current framework and guidelines for disease preparedness and response in Malaysia have resulted in the establishment of a health system that is both proactive and responsive to potential infectious disease outbreaks. Routine assessment of vaccines and novel therapeutic agents is essential in any infectious disease outbreak, with early treatment and interventions a useful tool in the arsenal [37]. It is important to assess and implement different resilience-building strategies considering the global spread of monkeypox. Across all strategies, a crucial element is the inclusive role of the community, the importance of public–private partnerships, and looking beyond the health sector. The future is uncertain, and efforts must be stepped up to prepare for any possibility in the future. What is certain is the important role of prepared and resilient health symptoms in mounting a response that manages the ill and protects all others, especially the vulnerable, in times of health crisis.

## Data Availability

The datasets used in the current study are not publicly available, but are available from the corresponding author on reasonable request.

## Abbreviations

COVID-19: Coronavirus Disease 2019
CPRC: National Crisis Preparedness and Response Center
IGMM: Interim Guidelines on Monkeypox Management
IMR: Institute for Medical Research
MKAK: National Public Health Laboratory
MOH: Ministry of Health
MPXV: Monkeypox virus
MSM: Men who have sex with men
PERHILITAN: Department of Wildlife Protection and National Parks of Peninsular Malaysia
PHEIC: Public Health Emergency of International Concern
WHO: World Health Organization
